# Determining the comparative pharmacodynamic equivalence of a non-invasive diagnostic test for patients with adrenal insufficiency using a randomised 2-way crossover trial: the STARLIT-3 study protocol

**DOI:** 10.1136/bmjopen-2025-112708

**Published:** 2026-02-05

**Authors:** Kathryn Date, Kathleen Baster, Sharon Caunt, Judith Cohen, Miguel Debono, Jane Fearnside, Trevor N Johnson, Peter Laud, Richard J Ross, Rosie Taylor, Charlotte Jane Elder

**Affiliations:** 1 Hull Health Trials Unit, Hull York Medical School, Hull, UK; 2 Statistical Services Unit, The University of Sheffield, Sheffield, UK; 3 Academic Directorate of Diabetes and Endocrinology, Sheffield Teaching Hospitals NHS Foundation Trust, Sheffield, UK; 4 School of Health and Related Research, The University of Sheffield, Sheffield, UK; 5 Translational Sciences Group, Certara UK Limited, Sheffield, UK; 6 Clinical Medicine, School of Medicine and Population Health, The University of Sheffield, Sheffield, UK

**Keywords:** Adrenal disorders, Paediatric endocrinology, Randomized Controlled Trial

## Abstract

**Introduction:**

Inadequate production of the essential stress hormone, cortisol, results in adrenal insufficiency (AI), which is associated with significant morbidity and mortality. The current standard diagnostic test for AI is the Short Synacthen Test (SST), but this is both invasive and resource-intensive, involving cannulation and blood sampling. A novel formulation, Nasacthin, has been developed in which the same Active Pharmaceutical Ingredient can be delivered intranasally, with the resultant glucocorticoid levels either measured in serum, or in saliva samples to render the test non-invasive, thus creating a potentially more cost-effective test. The Salivary Test of Adrenal Response to Liquid Intranasal Tetracosactide (STARLIT-3) study aims to determine the diagnostic utility of the test in patients with AI.

**Methods and analysis:**

STARLIT-3 is a randomised 2-way crossover trial which aims to collect data from 32 AI patients allocated to receive both Synacthen and Nasacthin in a random order across two study visits. Paired blood and saliva samples will be collected from participants at baseline, and then at 30 and 60 min after drug administration. Glucocorticoid levels in study samples will be quantified with the aim to determine whether the Nasacthin test is able to correctly diagnose patients with AI by estimating the positive percent agreement with the standard SST using serum cortisol at 30 and 60 min. Data on any reported harms and on the acceptability, usability and tolerability of the Nasacthin test will also be collected.

**Ethics and dissemination:**

The study and subsequent amendments have been reviewed and approved by South Central—Hampshire A Research Ethics Committee. Results will be published in peer-reviewed journals and presented at national and international conferences. Plans for dissemination of results to trial participants will be developed in collaboration with patient and public involvement and engagement groups.

**Trial registration number:**

ISRCTN26461337.

**STRENGTHS AND LIMITATIONS OF THIS STUDY**

- Robust study design that will produce data as agreed with the Medicines and Healthcare products Regulatory agency (MHRA) to support the regulatory approval of Nasacthin as part of a series of related studies.
- Randomised two-way crossover design enables direct within-participant comparison of Short Synacthen Test and Nasacthin test in adrenal insufficiency (AI) patients.
- Inclusion of detailed safety, acceptability, usability and tolerability data supports evaluation of practical implementation in clinical settings.
- Small sample size (32 participants completing both visits) limits statistical power to investigate any potential impact from factors such as ethnicity, body mass index, types of AI etc.
- Exclusion of children <4 years of age (unable to participate due to salivary collection techniques), and coryzal illness, hay fever etc. (which could potentially influence the absorption of nasal drug) could restrict applicability.

## Introduction

### Background and rationale

 Adrenal insufficiency (AI) is a potentially life-threatening endocrine disorder characterised by inadequate adrenal gland cortisol production due to endogenous or iatrogenic causes. Primary AI originates from adrenal gland dysfunction and direct impairment of cortisol production. The term central AI is associated with impaired production or action of adrenocorticotropic hormone (ACTH)^1^ and collectively describes both secondary AI (specifically related to pituitary defects) and tertiary AI (of hypothalamic origin). The most prevalent form is iatrogenic tertiary AI owing to sustained exogenous glucocorticoid use and resultant hypothalamic suppression.^2^

Diagnosis of AI is frequently delayed due to non-specific signs and symptoms at presentation. This risks the development of an acute life-threatening complication, adrenal crisis. Timely identification of AI is therefore essential.

In current clinical practice, the most performed diagnostic test for AI is the Short Synacthen Test (SST).^3 4^ This involves either intravenous (IV) or intramuscular injection of Synacthen (active ingredient tetracosactide), a synthetic analogue of ACTH (ACTH1-24), to simulate endogenous production from the adrenal cortex. The resulting serum cortisol response is quantified at 30±60 min. There is increasing demand for SSTs across all healthcare sectors^3 4^ due to increased glucocorticoid prescribing. The test is time-intensive, labour-intensive and resource-intensive, requiring skilled personnel with an associated inpatient tariff,^5^ and venesection which can be distressing and painful, especially for children.^6^,^8^

Salivary glucocorticoid measurement provides a non-invasive and more patient-friendly alternative to serum cortisol measurement and is increasing in adoption in clinical practice.^5 9^ A strong association between serum cortisol and salivary cortisol has been demonstrated,^10^,^14^ and salivary cortisol is not affected by interference from circulating cortisol-binding proteins, such as albumin or corticosteroid-binding globulin. Late-night salivary cortisol measurement is already recommended in the diagnosis of Cushing’s syndrome,^11^ and both basal and stimulated morning salivary cortisol levels pre-Synacthen and post-Synacthen have been investigated in both healthy and patient populations as a potential approach to AI diagnosis.^15^,^18^

Rapid enzymatic conversion of cortisol in saliva to cortisone due to expression of 11β-hydroxysteroid dehydrogenase type 2 in salivary glands means that the concentration of cortisone is typically 4 to 6 times higher than that of cortisol in saliva^10 19^ and is detectable at low serum cortisol levels.^13 14^ Salivary cortisone has been found to exhibit a better and more linear correlation with serum total cortisol than salivary cortisol^13 14 20^ and, as such, salivary cortisone is generally considered the preferred salivary biomarker for AI diagnosis.^9^ A recent NICE evidence review recommended further research on the clinical and cost-effectiveness of salivary cortisone/cortisol for AI diagnosis.^21^

The Nasacthin test has been developed as a non-invasive alternative to the SST stimulation test. The tests both use the same active pharmaceutical ingredient, tetracosactide, but in Nasacthin this is combined with a mucoadhesive agent, chitosan, to aid nasal absorption, and is delivered intranasally via a spray. Resultant glucocorticoid levels (cortisol and cortisone) can be measured in serum, but to render the test needle-free, saliva samples can be collected to quantify the adrenal response.

Nasacthin has been trialled over four open-label multi-arm sequence-randomised crossover pharmacodynamics studies^22^ across which a total of 70 doses of Nasacthin were administered to healthy males and children of both sexes. In these studies, it was found to be reliably absorbed, demonstrating a comparable plasma cortisol response to the IV SST at 60 min (the optimal timing of salivary glucocorticoid sampling following stimulation with IV Synacthen^17^), and well-tolerated with no serious adverse event (SAE) reports. Any AEs recorded were anticipated following nasal drug administration and were mild with full and rapid resolution.

If successfully adopted into clinical practice, Nasacthin would provide a simpler, non-invasive, easy-to-administer alternative to the SST, that could be carried out at the point of need. This would offer the potential to reduce the length of time to diagnosis, helping to reduce missed diagnoses and deaths from adrenal crisis, and wean patients from steroids in a more timely manner. The Nasacthin test has the potential to provide a more streamlined and cost-effective clinical care pathway, with particular benefit to low and middle-income countries where testing in conditions such as tuberculosis (TB) and AIDS is impacted by prohibitively high costs of Synacthen testing.^23^,^25^

The Salivary Test of Adrenal Response to Liquid Intranasal Tetracosactide (STARLIT-3) study aims to demonstrate a comparative diagnostic performance of Nasacthin in an AI patient population to the reference standard SST routinely used in clinical practice. The related STARLIT-2 study, which closed to recruitment in April 2025, aims to provide a parallel comparison of the SST and the Nasacthin test in healthy volunteers, including men, women and children.^26^

## Methods and analysis

This trial methodology is reported in accordance with the standard protocol items: recommendations for interventional trials (SPIRIT) reporting guidelines.^27^

### Trial aims and objectives

The STARLIT-3 study is designed to demonstrate that adrenal function testing with Nasacthin investigational medicinal product (IMP) can be used to correctly diagnose patients with AI at a comparable level to the standard IV Synacthen test, primarily at 30 min post-IMP administration, but also after 60 min, the optimal timepoint for salivary glucocorticoid sampling.^17^

Data on any harms or unintended effects associated with the use of Nasacthin will also be collected, along with qualitative data from focus groups and questionnaires to explore the acceptability, usability and tolerability of Nasacthin administration in study participants and by healthcare professionals. A further exploratory objective will compare the serum cortisol, salivary cortisol and salivary cortisone responses at 30 and 60 min following Synacthen versus Nasacthin administration.

### Trial design

This is a randomised two-way crossover trial involving patients with AI across two participating study centres, Sheffield Children’s NHS Foundation Trust (Sheffield, UK) and Sheffield Teaching Hospitals NHS Foundation Trust (Sheffield, UK). The crossover design will ensure that any differences observed between the two IMPs are derived from within-participant comparison.

### Eligibility criteria

The study aims to recruit patients (aged 4–75 years) with known AI, confirmed with either a waking salivary cortisone of <7 nmol/L, basal cortisol <150 nmol/L or peak on SST <250 nmol/L at time of diagnosis or since. An additional waking salivary cortisone test may be requested to confirm eligibility in instances where a previous glucocorticoid result cannot be obtained. Patients with a confirmed pathology compatible with a diagnosis of AI (eg, Congenital Adrenal Hyperplasia or autoimmune Addison’s disease) and a basal cortisol 150–299 nmol/L or SST peak 250–299 nmol/L will also be recruited if eligibility is confirmed by a waking salivary cortisone of <7 nmol/L.

Eligibility will be confirmed in accordance with exclusion criteria listed in box 1.

Box 1 **Trial exclusion criteria**

- Ongoing pregnancy.
- Use of oestrogen-containing hormonal contraception/hormone replacement therapy.
- Co-morbid condition requiring daily administration of a medication that interferes with the metabolism of glucocorticoids, including all oestrogens, loperamide, oral antifungals and opiates.
- Currently prescribed anti-epileptic medication, such as sodium valproate, phenytoin, clonazepam, nitrazepam, phenobarbital or primidone.
- Currently prescribed amphetamines, for example, lisdexamfetamine, dexamphetamine.
- Known and active protein-losing disorder, for example, enteropathy or nephrotic syndrome.
- Known clinical or biochemical evidence of hepatic or renal disease.
- Current uncontrolled active infection.
- Known or suspected alcohol dependence or drug misuse.
- Current smoker or vaper (or within 6 months of cessation).
- Recent (within last 1 week) liquorice ingestion (preparations containing glycyrrhizic acid only).
- History of known salivary gland or oral mucosa pathology or unable to produce a suitable salivary sample (eg, as a consequence of drugs that cause dry mouth).
- Previous severe allergic reaction or anaphylaxis, or adverse reaction to any antigen of adrenocorticotropic hormone (ACTH) or Synacthen.
- Participation in another clinical trial of an investigational or licensed drug or device within the last 3 months.
- Unable to comply with the requirements of the protocol.
- Any other significant medical or psychiatric condition that, in the opinion of the investigator, would preclude participation in the trial.

Exclusions for Nasacthin (nasal investigational medicinal product) visits only (visit should be rescheduled):

- Active nasal symptoms, including coryzal symptoms within the last week, active allergic rhinitis (hay fever) symptoms currently requiring medication, or heavy nosebleed within the previous 48 hours.

### Sample size

A sample size of 30 participants completing both study visits has been calculated using SAS PROC POWER to provide >90% power to show a positive percent agreement (PPA) of >80%, assuming a 5% alpha and a 1-sided exact test. The overall sample size has been increased to 32 completed participants to allow for up to two participants who are not identified as having AI by the SST being excluded from the PPA calculation to ensure at least 30 remain for inclusion in the main analysis. To account for predicted attrition, the recruitment target has been set at 41 participants.

Participants will be asked to return for a repeat visit or will be replaced if unable to produce the requisite samples for a visit (eg, due to cannula failure or inability to produce sufficient saliva). An absolute minimum of 25 pairs of samples from participants with confirmed AI (re-assessed as part of the trial using the SST) would provide 80% power to demonstrate PPA >80%, using serum cortisol at 30 min, for the final primary endpoint analysis.

### Recruitment

Potentially eligible patients with AI will be identified by clinical teams at participating sites and approached either during scheduled clinic appointments or by invitation letter or email. For potential participants under 16 years of age, the initial approach will be to a parent/legal guardian as proxy. Study posters with a QR code link to the study website will also be displayed in endocrinology outpatient clinics and endocrine daycare units. Expression of interest in trial participation can be made directly to the research team or online via a survey link accessed via the study information provided.

Age-appropriate study-specific information will be provided to interested participants and their families by the site research team in response to an expression of interest, or can be accessed via the study website https://hhtu.hull.ac.uk/starlit-3/#tab-10804. An additional Participant Information Leaflet has been developed specifically for parents/legal guardians. An Easy Read version is also available to support inclusion of patients with recognised learning impairment or additional needs who have the capacity to give consent.

Eligibility will be confirmed by the site Principal Investigator or other appropriately delegated clinician, and then informed consent will be received by an appropriately trained and delegated study clinician or research nurse prior to any participant-specific study-related activities.

### Informed consent

Informed consent will primarily be received during a real-time telephone or video call using remote electronic consent (e-Consent) via DocuSign, although face-to-face and postal options may be used if requested. Consent for all participants under 16 years of age must be provided by a parent/legal guardian with confirmed parental responsibility. Current versions of the consent forms can be found in onlinesupplemental materials 1  2. Child participants will be invited to sign a paper assent form to affirm agreement if deemed developmentally appropriate. Ongoing eligibility and consent will be verbally re-confirmed at each study visit.

Copies of all completed consent and assent forms will be uploaded into the study database to enable remote central monitoring of the consent process. Enrolment of consenting participants in the study database will automatically generate a unique pseudonymised participant ID number.

### Randomisation

All enrolled participants will be randomised sequentially via the purpose-built secure web-based data capture system with integrated randomisation function held on REDCap Cloud (RCC; www.redcapcloud.com). This is a two-way crossover trial in which participants will be randomised to receive both the test and comparator study IMPs (Nasacthin and Synacthen), one per study visit, in a pre-determined, randomly allocated order. Full details of the randomisation schedule will be held in RCC and will not be routinely accessible to the study team.

### Trial investigational medicinal product (IMP)

The test IMP for the study is Nasacthin (Huddersfield Pharmacy Specials, Huddersfield, UK) and the comparator IMP is Synacthen (Atnahs Pharma UK, Basildon, UK). Details of administration are outlined in table 1. Nasacthin (500 µg tetracosactide+chitosan) will be administered via a primed intranasal Mucosal Atomisation Device (DART300, Pulmodyne, Indiana, USA) (0.1 mL to each nostril). Synacthen will be administered as a slow IV bolus via the indwelling cannula.

**Table 1:** Trial investigational medicinal products

	Test IMP (Nasacthin)	Comparator IMP (Synacthen)
Constituents	500 µg tetracosactide + chitosan	250 µg tetracosactide
Administration route	Intranasal (via MAD)	Intravenous (via cannula)
Dose	0.2 mL (0.1 mL to each nostril)	250 µg (1 mL) for adults 145 µg/m^2^ for children

IMP, investigational medicinal product; MAD, Mucosal Atomisation Device.

The Nasacthin 500 µg dose is derived from previous pharmacodynamic work demonstrating a comparable plasma cortisol response to the SST at 60 min.^22^ Synacthen will be administered at the standard doses used for routine SST testing, that is, 250 µg (1 mL) in adult participants and 145 µg/m^2^ in children, as recommended in the British National Formulary for children (BNFc).^28^ The same doses have been adopted in the closely related STARLIT-2 study^26^ (which closed to recruitment in April 2025) to ensure directly comparable safety and efficacy data.

### Trial procedures

**Figure 1:**
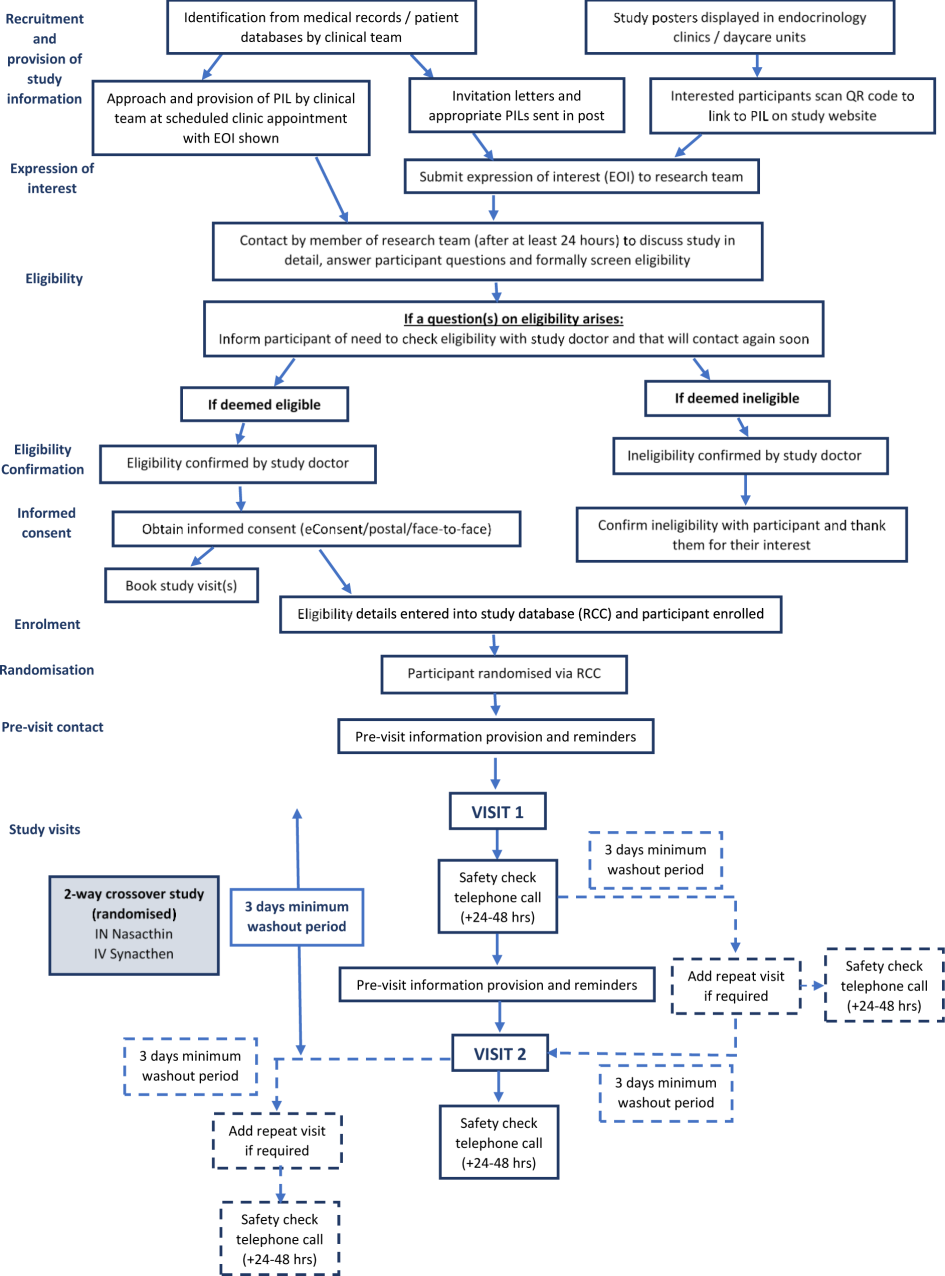
Flow diagram of participant trial procedures.

Trial participation is outlined in figure 1. Participants will attend two study visits with a minimum 3 day washout period between each visit to ensure elimination of any previous tetracosactide received and potential resultant effects on endogenous glucocorticoid levels. This was calculated based on the half-lives for both tetracosactide (34 min) and cortisol (120 min) from our previous studies^22^ and those reported in the literature.^29 30^ Five half-lives were taken as the period to minimal exposure/return to baseline (approximately ten hours), thus 3 days is thought to be a suitable washout period.

Due to potential interference with glucocorticoid measurement and in accordance with standard practice for an adrenal function test, participants will be asked to withhold their regular steroid medications taken for management of their AI prior to each visit (see table 2). This will be discussed with participants prior to consent and will be approved by their treating clinician prior to trial participation. Participants will bring their steroid medications to the study visit and take their next dose as soon as possible after collection of the final study samples.

**Table 2:** Trial steroid medication omission guidance

Steroid type	Participant instruction
Oral Hydrocortisone	Omit the night/evening before and morning of each visit
Oral Prednisolone	Omit doses for 24 hours before visit
Oral Dexamethasone	Omit normal doses for 72 hours prior to each visit
Oral Fludrocortisone	Withhold administration the morning of the visit
Steroid creams, asthma inhalers, steroid nose sprays, steroid eye drops and all other non-oral steroids	Withhold administration the night before and morning of each visit
Parental steroids (except injectable hydrocortisone)	Can only participate 12 weeks after last injection

Participants will be contacted 1 week prior and 2 days prior to each visit to confirm attendance, provide important reminders regarding omission of steroid medications and check essential visit-specific exclusions. Any participants taking increased doses of steroids due to current or recent illness (‘stress dosing’) will be advised to reschedule the visit.

Participants will be asked to abstain from liquorice ingestion within the last week (preparations containing glycyrrhizic acid only due to its inhibitory effect on 11β-hydroxysteroid dehydrogenase type-2), and alcohol consumption and recreational drug use for 24 hours. Any Nasacthin visits will be rescheduled if a participant has experienced coryzal symptoms in the last week, a heavy nosebleed in the previous 48 hours, or has active allergic rhinitis (hay fever) symptoms requiring medication, due to their unconfirmed effects on nasal drug absorption.

Study visits will be scheduled to commence between 08:00 and 09:00 with the aim to collect baseline sample measurements while cortisol levels are close to their natural physiological peak to aid interpretation. A confirmed negative pregnancy test is required for all participants of childbearing potential before study activities can take place, due to the potential for altered cortisol levels and as per safety advice to avoid Synacthen administration in pregnancy where possible.

At the first study visit, basic demographic data, and height and weight measurements will be collected. The height and weight measurement will be repeated at subsequent visits for any child participants who have more than 3 months between visits.

At the start of each visit, a set of baseline clinical observations (temperature, pulse rate, blood pressure and respiratory rate) will be taken, and the date and time of last administration for all regular steroid medications will be checked and documented. The visit will be rescheduled if the participant is unwell or has not adhered to the steroid medication omission guidance.

Following IV cannulation, participants will rest supine for a minimum of 20 min. Ten minutes prior to commencing sample collection, participants will be asked to rinse their mouth thoroughly with water to minimise sample contamination. A pair of baseline blood and saliva samples will then be collected. Participants will be asked to refrain from eating or drinking within 30 min prior to baseline sample collection.

All whole blood samples will be collected from the indwelling cannula into serum separating tubes. Samples will be gently mixed and left to clot for 30–60 min at room temperature, before centrifuging at 1300 x*g* for 10 min. Resulting serum will be aliquoted into cryovials for storage. All saliva samples will be collected by passive drool into a Saliva Collection Aid (Salimetrics, Pennsylvania, USA) and may be centrifuged at 1000 rpm to reduce any bubbles if necessary. All samples will then be transferred to −80°C freezer storage prior to shipment to a central laboratory for analysis.

Study IMP will be administered immediately after baseline sample collection (as per table 1), in accordance with the allocated randomised sequence. Due to the effect of stress on cortisol levels, re-cannulation following cannula failure post-IMP administration is not permitted.

Further paired blood and saliva samples will then be taken at 30 min and 60 min post-IMP administration, with participants rinsing their mouth with water ten minutes prior to each saliva sample collection. Eating or drinking (except water) will not be permitted during the study sampling period. Participants will be advised to take their next doses of all regular steroid medications as soon as sample collection is completed.

All participants will receive a follow-up telephone call 24–48 hours (or next available working day) after each visit to check for the emergence of coryzal symptoms or any AEs.

In instances where individual study visits need to be repeated (eg, if requisite study samples cannot be collected), the same IMP will be administered at the repeated visit to maintain the allocated IMP sequence.

### Trial outcomes and assessments

Analysis of participant study samples for glucocorticoid levels will be performed at a central laboratory using liquid chromatography with tandem mass spectrometry (LC-MS/MS) assays to quantify the levels of serum cortisol, and salivary cortisol and cortisone. Optional consent for submission of any residual samples to a biorepository following completion of analysis will be obtained as part of the main consent process.

Cortisol levels at baseline and 30 min post-administration of Synacthen and Nasacthin will be used to determine the primary study endpoint, the PPA, which is defined as the proportion of participants known to have AI (as detected by the SST) correctly diagnosed by the Nasacthin test using the same threshold. This will also be measured as a secondary endpoint using the baseline and 60 min serum cortisol results as the current sampling timepoints for the SST. For the exploratory objective, serum cortisol, salivary cortisol and salivary cortisone levels at baseline and at 30 min and 60 min post-administration of Nasacthin and Synacthen will be used to produce summaries of the comparative responses.

Details of all harms or unintended effects occurring during trial participation will be collected and used to determine the frequency of AEs, SAEs and suspected unexpected serious adverse reactions (SUSARs) by treatment arm. Participants will be closely monitored for any AEs occurring following IMP administration, particularly in the first 30 min for any sign of hypersensitivity reaction. The follow-up telephone call will be used as a safety check for participants to report any AEs that may have occurred since the visit. Any harms or unintended effects that are considered serious will be logged electronically via the study database within 24 hours of first knowledge and reported as appropriate.

Participant evaluation of the acceptability and tolerability of Nasacthin administration will be sought using a short non-validated questionnaire to be completed at the end of each visit, and an additional questionnaire at the final study visit to compare the IV and nasal tests. Participants will also be invited to take part in a focus group for further exploration of these aspects.

At the completion of trial recruitment, healthcare professionals at participating sites will be asked to complete an anonymous online questionnaire on the comparative acceptability of administration of the two tests. To understand the utility and future barriers to adoption of the Nasacthin test, key stakeholders (eg, endocrinologists (paediatric and adult), nurses, clinicians from other heavy steroid-using specialties, pharmacists, biochemists, primary and community care providers, commissioners and service managers etc.) will be invited to attend focus groups to discuss the usability and practicalities of the different testing methods.

### Data collection and management

All information collected about participants during the trial will remain confidential and will be held in accordance with General Data Protection Regulation (GDPR) 2018. All study participants will be automatically assigned a unique identifying number at the point of enrolment onto the study database. All Hull Health Trials Unit (HHTU) data systems are within scope of the HHTU NHS Data Security and Protection Toolkit.

Study data will be collected and stored in a purpose-built, secure online electronic data capture system with integrated randomisation function and role-based user access held within the HHTU instance of RCC to enable central monitoring of trial recruitment and study visit completion. The database will be built and validated according to study-specific requirements outlined in a Data Management Plan agreed by the CI, Sponsor and HHTU. All activity within RCC is subject to a full user audit, with both automated and manual checks to monitor data quality and completeness according to a sponsor-approved monitoring plan.

Individual participant data will mostly be inputted directly into electronic case report forms held within the study database. Information on all AEs, SAEs and SUSARs occurring during the trial will be collected in the study database and coded using the medical dictionary for regulatory activities (MedDRA) prior to database lock.

Participant evaluation of intervention acceptability and tolerability will be captured on paper questionnaires, and these data will then be entered at site onto RCC. The views of healthcare professionals involved in the study will be captured anonymously via a survey completed and held within RCC. Focus groups will be audio or video recorded and transcribed verbatim, and resulting transcripts labelled with unique identifiers ready for analysis. Audio/video recordings and the resulting anonymised transcripts will be held in the secure HHTU instance of Box.com collaborative cloud file storage system. Original audio/video recordings will be destroyed once transcription is completed and allocation of unique identifiers has been assigned.

Glucocorticoid results from analysis of serum and saliva samples received from the central lab will be uploaded in a lab report to a dedicated (user access-restricted) secure location on Box. The HHTU data team will perform an initial check prior to data entry into RCC and an independent second check in RCC once data has been added.

eConsent signatures and associated data will be captured using DocuSign cloud-based signature platform. Although copies of consent forms will be stored on Box, they will not be exported as part of the final trial dataset.

The database will be locked to obtain the final dataset after trial completion and resolution of all data queries. A copy of the final trial dataset and notification of completion of trial visits will be sent to the Sponsor before the randomisation list can be released to HHTU prior to statistical analysis. Locked study datasets will be exported from RCC and transferred via a secure folder in Box to the trial statistician at the University of Sheffield and the qualitative researcher at Sheffield Teaching Hospitals NHS Foundation Trust for analysis in compliance with GDPR 2018. A copy of each individual site’s data will be sent to the site, and a copy of the final trial dataset will be archived by HHTU.

### Statistical analysis

Statistical analysis will follow the methodology detailed in the study-specific statistical analysis plan.

#### Blind data review

While no formal interim analysis is planned, the trial statistician will perform a quality control check on cortisol and cortisone values derived from each batch of study samples throughout the data collection phase and prior to provision of the final dataset. Individual visit data will be treated as completely independent (no within-participant linkage across visits) with original participant ID numbers removed from the dataset to preserve blinding. Spaghetti plots of all timepoints within each visit will be created and grouped by endpoint—serum cortisol, salivary cortisol, salivary cortisone. Any anomalies noted will be explored and resolved prior to database lock.

#### Final analysis

The full analysis set (FAS) will include all participants who have had at least one dose of study treatment (Nasacthin or Synacthen) and have at least one observation of serum cortisol or salivary cortisone. This is a modified intention-to-treat analysis set as participants who do not receive any treatment or who do not provide any cortisol/cortisone observations will not be included in the analysis. This analysis set will be used for the analysis of all efficacy data.

The Per-protocol Analysis set will include all participants who are deemed to have no important protocol deviations that could interfere with the primary objectives of the study. This is a sub-population of the FAS.

The safety analysis set (SAS) will include all participants who have received at least one dose and will be used for the analysis of safety data (AEs).

Continuous data will be summarised using n, mean, SD, median, minimum and maximum. Lower and upper quartiles may also be calculated as necessary. Demographics and any baseline characteristics will be summarised overall for the SAS. Medical history, which will not be coded, will be listed but not summarised.

All available data will be included in each analysis. The key timepoint for analysis is the 30 min post-administration of either IMP. A participant does not need to have provided data at every time point after every treatment to be included in the analysis, therefore it is possible that the number of participants included in each analysis may vary.

All study participants will be re-assessed for AI as part of the trial using the SST. A diagnostic serum cortisol cut-off of ≤429 nmol/L will be the reference standard result, which will be used for the calculation of the PPA. The proportion will be calculated together with a 95% exact CI using the Clopper-Pearson method.

Analysis for the primary objective will include the subset of the FAS who fail the SST (ie, have AI) and have provided a serum cortisol sample at 30 min post-Nasacthin dose; any participants in the FAS who pass the SST or who have not provided a post-Nasacthin serum cortisol sample will be summarised.

The PPA calculation for the primary outcome analysis will then be repeated using serum cortisol levels obtained on the Nasacthin test at 60 min compared with the SST result at both 30 and 60 min.

The safety of Nasacthin will be assessed by reporting on the frequency of AEs, SAEs and SUSARs by treatment. AEs, SAEs and SUSARs will be summarised by treatment received, system organ class and preferred term. If there are very few AEs (eg, ≤8), then they will be listed only. Graphical summaries will be considered if the number of AEs is large.

For the exploratory objective, the serum cortisol, salivary cortisol and salivary cortisone responses at 30 and 60 min after each of the active treatments will be summarised using n, mean, SD, median, minimum and maximum.

#### Sensitivity analysis

If participants report coryzal symptoms starting within 24 hours of the visit, these will be recorded and summarised. A sensitivity analysis may be carried out for the primary objective excluding observations taken at these visits if there is a concern that inflamed mucus membranes may impact how the nasal drug works.

### Qualitative research analysis

Analysis of participant and healthcare professional perspectives on acceptability, tolerability and usability of the Nasacthin test will be reported using descriptive statistics by calculating rates and percentages. Open-ended text boxes will be analysed using content analysis and coded into a range of categories using NVivo. Thematic analysis on transcripts from participant and stakeholder focus groups will be completed using NVivo.

### Patient and public involvement and engagement (PPIE)

Through consultation with the Lay ADvice on Diabetes and Endocrine Research (LADDER) patient and public involvement and engagement (PPIE) group at Sheffield Teaching Hospitals NHS Foundation Trust and PPIE groups at Sheffield Children’s Hospital, cohorts of adult and paediatric patients with experience of the SST and/or salivary tests were convened to explore the unmet needs in greater depth and develop the research design.

Subsequent meetings with the LADDER group and additional Sheffield Children’s Hospital PPIE groups have been used to facilitate the co-production of trial documentation. The study team will provide updates on trial progress and further input will be sought from these groups to discuss any delivery issues, topic guide development for the participant and stakeholder focus groups and assist in plans for dissemination of results.

Additionally, two PPIE representatives serve as key members of the independent Trial Steering Committee to provide valuable patient perspective.

## Ethics and dissemination

### Regulatory approvals

The trial protocol and subsequent amendments have been reviewed and approved by the South Central-Hampshire A Research Ethics Committee (REC ref. 24/SC/0102) and by the Medicines and Healthcare Products regulatory Agency (MHRA) and HRA. The ISRCTN registry will be updated in accordance with any amendments to the study.

### Trial oversight

Sheffield Children’s NHS Foundation Trust is the study sponsor, and HHTU is responsible for trial implementation and management and will perform central and site monitoring in accordance with a sponsor-approved trial monitoring plan.

A trial management group (TMG) has been convened and meets regularly to oversee trial delivery and operations. An independent Trial Steering Committee (TSC), comprising two expert clinicians, a statistician, a trials methodologist and two PPIE representatives, will provide overall trial supervision and advice on behalf of the sponsor and project funder and hold executive power on key trial-related decisions. An independent data monitoring and ethics committee (DMEC), comprising two expert clinicians and a statistician, will monitor progress, review efficacy and safety data, and make recommendations to the TSC on study conduct and continuation where necessary.

### Safety considerations

Participants will be monitored for any AEs throughout their study visit and checks will be made during follow-up telephone calls. Any symptoms known to be associated with nasal drug administration will not be reported as AEs unless persistent (defined as still present 30 min after IMP administration).

SAEs are not anticipated during the trial due to the well-established safety profiles of the main IMP ingredients, that is, tetracosactide and chitosan. There will be a robust SAE reporting process in place, with reporting via the study database and automatic alerts to the study team.

The AE reporting period begins from the point of consent and ends 48 hours after the final trial visit. Participants will be advised to contact the study team if they experience any AEs within this period.

The occurrence of one possibly related serious AE or two possibly related severe AEs will trigger trial stopping criteria. The trial can only be restarted following discussion with the DMEC and MHRA approval of a substantial amendment.

If a possibly related serious or severe AE occurs in any individual participant, they should not receive any subsequent IMP and will be withdrawn from the trial.

### Dissemination

Trial results will be published in peer-reviewed journals and disseminated via conference presentations at national and international meetings in accordance with the study publication plan as approved by the TMG. Authorship will be determined according to the International Committee of Medical Journal Editors (ICMJE) guidelines.^31^ Plans for dissemination of results to trial participants and the public will be developed in consultation with PPIE groups.

### Data availability

Trial data will not be released for sharing prior to the first publication of primary endpoint analysis in an open-access peer-reviewed journal and conclusion of marketing authorisation/commercialisation discussions with the MHRA and potential licensing partners.

Final anonymised clinical study datasets and meta-data will be prepared and stored in an appropriate format to enable discoverability and sharing within the national data repository available to Medical Research Council (MRC) researchers, UK Data Service (UKDS) ReShare, but with an appropriate embargo on release. Requests for access to the dataset will be managed via ReShare’s established processes and will be enabled via a Data Sharing Agreement.

### Protocol version

This paper is based on Protocol V.1.10; 1 August 2025.

### Current trial status

The STARLIT-3 study opened to recruitment on 5 February 2025 and has an 14-month recruitment window.

## Supplementary material

10.1136/bmjopen-2025-112708 online supplemental file 1

10.1136/bmjopen-2025-112708 online supplemental file 2
