# Correction: Determining the comparative pharmacodynamic equivalence of a non-invasive diagnostic test for patients with adrenal insufficiency using a randomised 2-way crossover trial: the STARLIT-3 study protocol

**DOI:** 10.1136/bmjopen-2025-112708corr1

**Published:** 2026-04-02

Date K, Baster K, Caunt S, *et al*. Determining the comparative pharmacodynamic equivalence of a non-invasive diagnostic test for patients with adrenal insufficiency using a randomised 2-way crossover trial: the STARLIT-3 study protocol. *BMJ Open* 2026;16:e112708. doi: 10.1136/bmjopen-2025-112708

The article has been corrected following its online publication.

The authors wish to inform readers that co-author Trevor N Johnson (Translational Sciences Group, Certara UK Limited, Sheffield, UK) was inadvertently omitted from the author list. TNJ contributed to data provision, data interpretation and references which were integral to the study methodology in the calculation of the required wash-out period for the IMP and advised on the drafting of this section of the manuscript.

