# Supplementary material for: Determining the comparative pharmacodynamic equivalence of a non-invasive diagnostic test for patients with adrenal insufficiency using a randomised 2-way crossover trial: the STARLIT-3 study protocol
Source: BMJ Open. 2026 Feb 5;16(2):e112708. doi: 10.1136/bmjopen-2025-112708 (PMC12878249; doi:10.1136/bmjopen-2025-112708)
Supplement: online supplemental file 1 [file bmjopen-16-2-s001.pdf]

<< insert site logo >>

STARLIT-3 INFORMED CONSENT FORM

ADULT PARTICIPANTS [eCONSENT]

Study Title: Salivary Test of Adrenal Response to Liquid Intranasal Tetracosactide – Study **3** (STARLIT-3)
Name of Researcher:
Participant ID Number:

		*Please **initial** each box below*
1.	I confirm that I have read and understood the information sheet dated XX.XX.XXXX (version X.X) for the above study.	
2.	I confirm that I have had the opportunity to consider the study information, ask questions and have had these answered satisfactorily.	
3.	I understand that my participation is voluntary and that I am free to withdraw at any time without giving any reason, without my medical care or legal rights being affected. I understand that any remaining samples will be destroyed at my request provided the samples have not been transferred to the laboratory for analysis.	
4.	I understand that medical records and data collected during the study may be accessed and looked at by individuals from the NHS Trust research team, Hull Health Trials Unit and regulatory authorities, where it is relevant to my taking part in this research.	
5.	I agree to the secure transfer, storage and use of paper and electronic personal information for the purposes of this study to Hull Health Trials Unit and the University of Sheffield.	
6.	I understand that any information that could identify me will be kept strictly confidential and that no personal information will be included in the study report or other publication.	
7.	I understand that the information collected about me may be used to support other ethically approved future research projects and may be shared anonymously with other researchers.	
8.	I agree to take part in the above study.	
IF APPLICABLE (*for Person Of Child Bearing Potential (POCBP only)*		
9.	I agree to provide a urine sample for a pregnancy test at each study visit.	

Sheffield Children's NHS NHS Foundation Trust · University of Sheffield · University of Hull | Hull Health Trials Unit · UKRI Medical Research Council

OPTIONAL (*you do not have to consent to these points in order to participate in the main study*)<br>*Please **initial** the appropriate box below*		
10.	I agree to my General Practitioner (GP) being informed of my participation in this study.	YES<br><br>NO
11.	I am happy to be contacted at a later date to take part in a focus group about the study.	YES<br><br>NO
12.	I am happy to be contacted to take part in future research.	YES<br><br>NO
13.	I give consent for any of the blood and saliva samples that I give that are used as part of this study to be used in future ethically approved research projects.	YES<br><br>NO
14.	I wish to be provided with a summary of the research findings once the study is complete.	YES<br><br>NO

**Participant**		
Name (*please print*)	Date	Signature

**Person receiving consent**			
Name (*please print*)	Professional Registration Number	Date	Signature

When completed: 1 copy (original) for Investigator Site File; 1 copy for participant; 1 copy for clinical record; 1 copy for HHTU
