# Supplementary material for: Determining the comparative pharmacodynamic equivalence of a non-invasive diagnostic test for patients with adrenal insufficiency using a randomised 2-way crossover trial: the STARLIT-3 study protocol
Source: BMJ Open. 2026 Feb 5;16(2):e112708. doi: 10.1136/bmjopen-2025-112708 (PMC12878249; doi:10.1136/bmjopen-2025-112708)
Supplement: online supplemental file 2 [file bmjopen-16-2-s002.pdf]

<< insert site logo >>

STARLIT-3 INFORMED CONSENT FORM
PARENT / LEGAL GUARDIAN [eCONSENT]

Study Title: **S**alivary **T**est of **A**drenal **R**esponse to **L**iquid **I**ntranasal **T**etracosactide – Study **3** (STARLIT-3)
Name of Researcher:
Participant ID Number:

		*Please **initial** each box below*
1.	I confirm that I have read and understood the information sheet dated XX.XX.XXXX (version X.X) for the above study.	
2.	I confirm that I have had the opportunity to consider the study information, ask questions and have had these answered satisfactorily.	
3.	I understand that my child's participation is voluntary and that I am free to withdraw my child at any time without giving any reason, without my child's medical care or legal rights being affected. I understand that any remaining samples belonging to my child will be destroyed at my request provided the samples have not been transferred to the laboratory for analysis.	
4.	I understand that medical records and data collected during the study may be accessed and looked at by individuals from the NHS Trust research team, Hull Health Trials Unit and regulatory authorities, where it is relevant to my child taking part in this research.	
5.	I agree to the secure transfer, storage and use of paper and electronic personal information for the purposes of this study to Hull Health Trials Unit and the University of Sheffield.	
6.	I understand that any information that could identify my child will be kept strictly confidential and that no personal information will be included in the study report or other publication.	
7.	I understand that the information collected about my child may be used to support other ethically approved future research projects and may be shared anonymously with other researchers.	
8.	I agree to my child taking part in the above study.	
IF APPLICABLE (*for Person Of Child Bearing Potential (POCBP only)*)		
9.	I agree for my child to provide a urine sample for a pregnancy test at each study visit.	

OPTIONAL (*you do not have to consent to these points in order for your child to participate in the main study*)<br>*Please **initial** the appropriate box below*		
10.	I agree to my child's General Practitioner (GP) being informed of their participation in this study.	YES<br><br>NO
11.	I am happy to be contacted at a later date for me and my child to take part in a focus group about the study.	YES<br><br>NO
12.	I am happy to be contacted about my child taking part in future research.	YES<br><br>NO
13.	I give consent for any of the blood and saliva samples that my child gives that are used as part of this study to be used in future ethically approved research projects.	YES<br><br>NO
14.	I wish to be provided with a summary of the research findings once the study is complete.	YES<br><br>NO

**Parent / Legal Guardian**	
Name of participant (*please print*)	
Name of participant's parent or legal guardian (*please print*)	
Relationship to participant	
Date	Signature

**Person receiving consent**			
Name (*please print*)	Professional Registration Number	Date	Signature

When completed: 1 copy (original) Investigator Site File; 1 copy for parent / legal guardian; 1 copy for clinical record; 1 copy for HHTU
